# Landscape of m^6^A Modification in Testicular Tissues of Normal and Cryptorchid Tianhua Mutton Sheep

**DOI:** 10.3390/ani16213460

**Published:** 2026-11-02

**Authors:** Jiamei Liu, Weili Liang, Wanhong Li, Xiangpeng Yue, Fadi Li

**Affiliations:** State Key Laboratory of Herbage Improvement and Grassland Agro-Ecosystems, Key Laboratory of Grassland Livestock Industry Innovation, Ministry of Agriculture and Rural Affairs, Engineering Research Center of Grassland Industry, Ministry of Education, College of Pastoral Agriculture Science and Technology, Lanzhou University, Lanzhou 730020, China; liujm2023@lzu.edu.cn (J.L.); liangwl2540@163.com (W.L.); lexp@lzu.edu.cn (X.Y.); lifd@lzu.edu.cn (F.L.)

**Keywords:** cryptorchidism, m^6^A, MeRIP-seq, RNA-seq, Tianhua mutton sheep, testis

## Abstract

Cryptorchidism is a common male reproductive disorder generally characterized by spermatogenic failure. Nevertheless, the molecular mechanisms underlying infertility induced by cryptorchidism in sexually mature rams remain insufficiently characterized. N^6^-methyladenosine (m^6^A), the most prevalent internal RNA modification, plays a dynamic role in testicular development and spermatogenesis. In the present study, testicular tissues collected from normal and cryptorchid Tianhua mutton sheep were used to construct a transcriptome-wide m^6^A methylation atlas of the testis. Histological observation showed significantly reduced numbers of Sertoli and Leydig cells and diminished seminiferous tubule diameters in cryptorchid testes. RNA sequencing (RNA-seq) results demonstrated the dysregulated tissue-level mRNA expression of m^6^A regulators in cryptorchid testes. Methylated RNA immunoprecipitation sequencing (MeRIP-seq) revealed 2080 differentially methylated m^6^A peaks, with 1449 hypermethylated and 631 hypomethylated m^6^A peaks in cryptorchid testes. The majority of these peaks were concentrated in the CDS region, followed by the stop codons and 3′ UTR. The integrative analysis of MeRIP-seq and RNA-seq data revealed that differentially expressed and m^6^A methylated genes were enriched in the apoptosis, AMPK, FoxO, TNF, and MAPK signaling pathways. Seven candidate genes, including *RRM2*, *GTSE1*, *ATM*, *GORAB*, *INSR*, *HSPA1L*, and *MAPK9* were screened. In conclusion, this study identified differences in the mRNA abundance of m^6^A regulators and regional m^6^A enrichment between normal and cryptorchid sheep testes. These tissue-level differences may partly reflect changes in cellular composition. The findings provide a basis for further investigation of the potential involvement of m^6^A regulation in cryptorchidism-associated spermatogenic impairment.

## Simple Summary

Cryptorchidism, in which one or both testes fail to descend normally, is a common cause of male infertility. However, the underlying biological changes remain unclear. This study aimed to investigate how cryptorchidism affects m^6^A modification on RNA. Testicular tissues from normal and cryptorchid nine-month-old Tianhua mutton sheep were compared. Cryptorchid testes had fewer Sertoli and Leydig cells, as well as narrower seminiferous tubules. The mRNA expression of m^6^A regulators and 2080 m^6^A methylation peaks differed between cryptorchid and normal testes. Further analysis identified 7 candidate genes linked to apoptosis, cell cycle regulation, DNA repair, stress response, and spermatogenic dysfunction. This work improves our understanding of male infertility and may inform future research aimed at improving reproductive health in livestock.

## 1. Introduction

Tianhua mutton sheep is a newly developed breed for both mutton and fine-wool production, derived from the crossbreeding of South Africa mutton Merino rams with Gansu alpine fine-wool ewes. This breed is distinguished by excellent mutton and wool quality, high fertility, and strong adaptability to cold, high-altitude environments. It is well suited to various feeding systems, including semi-pastoral/semi-intensive and fully intensive management systems [1]. Optimizing semen quality is critical for enhancing the reproductive performance of breeding rams. Normal testicular development provides the structural and hormonal environment essential for spermatogenesis and is therefore a fundamental determinant of male fertility.

Cryptorchidism is a common congenital reproductive disorder in mammals, defined by the failure of one or both testes to descend into the scrotum, resulting in their retention within the abdominal cavity or inguinal canal [2]. The incidence of cryptorchidism in sheep is generally low, ranging from 0.5% to 1% in breeds such as Sarda sheep [3,4,5,6]. However, it can be as high as 10–23% in certain breeds (e.g., Karagouniko) or inbred populations [7,8]. The occurrence of cryptorchidism is influenced by both genetic and environmental factors. To date, only a few genetic risk factors have been identified in humans and animal models. For example, insulin-like peptide 3 (INSL3) and its receptor, relaxin family peptide receptor 2 (RXFP2), play key roles in regulating the growth and development of the testicular cords [9,10]. In addition, environmental factors can increase the incidence of cryptorchidism [11].

As a natural model of prolonged heat exposure, cryptorchid testes are chronically subjected to supraphysiological temperatures, which impair spermatogenic epithelial function and markedly increase the risk of infertility [12]. Study has shown that compared with normal individuals, bilateral cryptorchid boars exhibit reduced testicular volume and decreased germ cell numbers [13]. Spermatogenic dysfunction in adult cryptorchid males primarily results from impaired self-renewal and differentiation of spermatogonia [14]. In rams, cryptorchidism disrupts the negative feedback loop regulating LH and FSH secretion, leading to a hormonal imbalance [15]. Elevated local testicular temperature can activate the p53 signaling pathway, thereby inducing germ cell apoptosis [16]. Nevertheless, the specific pathological mechanisms by which cryptorchidism leads to spermatogenic failure in sexually mature rams remain poorly understood, and few studies have examined the epigenetic regulatory network underlying cryptorchid-induced testicular pathology.

*N*^6^-methyladenosine (m^6^A) is the most abundant internal modification in eukaryotic mRNA. m^6^A deposition is mediated by methyltransferases, including the METTL3–METTL14 complex and METTL16, with WTAP functioning as a regulatory component of the METTL3–METTL14 complex [17,18,19]. The modification can be removed by the demethylases FTO and ALKBH5 [20,21]. Reader proteins, including members of the YTHDF family and IGF2BP1/2/3, recognize m^6^A-modified RNA and influence its fate [22,23,24]. Together, these regulators influence mRNA splicing, nuclear export, translation, and stability, contributing to post-transcriptional control of gene expression [22]. Accumulating evidence indicates that m^6^A modification plays a critical regulatory role in diverse biological processes, including animal development, immunity, and reproduction. Environmental stressors, such as DEHP exposure, elevate global testicular m^6^A levels, alter FTO and YTHDC2 expression, and induce testicular toxicity [25]. In livestock, an integrated analysis of m^6^A methylome and transcriptome sequencing data has identified four m^6^A-marked genes (*PLK4*, *PTEN*, *EGR6*, and *PSME6*) associated with testicular development and spermatogenesis regulation in Xia Nan cattle [26]. In mice, the m^6^A reader protein IGF2BP1 supports spermatogonial proliferation by stabilizing *Akt2* mRNA [27]. IGF2BP1 and AKT2 are down-regulated in testes of patients with cryptorchidism and oligozoospermia or azoospermia [28].

The m^6^A methylation landscape and its regulatory role in cryptorchid sheep remain uncharacterized. In this study, testicular tissue samples from cryptorchid (CR) and normal (control) Tianhua mutton sheep were collected. By using methylated RNA immunoprecipitation sequencing (MeRIP-seq) and RNA sequencing (RNA-seq), the differences in m^6^A modification patterns and gene expression between cryptorchid and normal testes were systematically analyzed. These findings provide a valuable foundation for understanding the role of m^6^A modifications in male reproduction.

## 2. Materials and Methods

### 2.1. Animals and Sample Collection

Testicular samples were collected from nine-month-old Tianhua mutton sheep (three congenital bilateral cryptorchid and three normal). Each lamb was housed under the same diet and management conditions at Minqin Defu Agriculture Co., Ltd. (Wuwei, China) similar to a previously described feeding pattern [29]. In the cryptorchid lambs, both testes were retained within the abdominal cavity. Following surgical removal from either the scrotum or abdominal cavity, the testes were weighed, and testicular tissues were immediately collected. One portion of each sample was snap-frozen in liquid nitrogen, and the other was fixed in testicular tissue fixative (Servicebio, Wuhan, China).

### 2.2. Histological Analysis

After fixation, testicular tissues were dehydrated in an alcohol gradient, cleared with xylene, and subsequently embedded in paraffin. Sections of 4 μm thickness were prepared and stained with hematoxylin–eosin. Images were acquired with an Olympus IX73 microscope (Olympus, Tokyo, Japan). The diameter of seminiferous tubules and Sertoli/Leydig cell numbers were analyzed by using cellSens software (version 3.2, Olympus, Tokyo, Japan). Sections were magnified 400×, and at least 10 seminiferous tubules from each sample were randomly selected per section for counting Sertoli cells, and the counts were averaged for each sample. Leydig cells within the triangular interstitial spaces surrounding nearly round seminiferous tubules were counted, with at least 10 triangular interstitial spaces quantified per sample [30]. The diameter of seminiferous tubules was measured under magnified 200×, and at least 30 nearly round seminiferous tubules were randomly selected per sample. The diameters were measured and averaged for each sample prior to between-group comparison. Each group comprised three biological replicates (*n* = 3).

### 2.3. m^6^A Quantification by LC–MS/MS

Three cryptorchid and three normal testicular samples were used for LC–MS/MS and subsequent MeRIP-seq and RNA-seq. The m^6^A content in the total RNA of the testis was quantified by LC–MS/MS at MetWare Biotechnology Co., Ltd. (Wuhan, China). Total RNA was isolated and purified by using TRIzol reagent. One microgram of total RNA with concentration >50 ng/μL, RIN value > 7.0, and OD_260_/_280_ > 1.8 was digested into nucleosides in S1 nuclease, phosphodiesterase I, and alkaline phosphatase at 37 °C. After chloroform extraction, the aqueous phase was collected for analysis on an UPLC–ESI–MS/MS system (UPLC, ExionLC^TM^ AD, SCIEX, Framingham, MA, USA; MS, Applied Biosystems 6500 Triple Quadrupole, SCIEX). The effluent was alternatively connected to an ESI–QTRAP–MS (550 °C, ESI+, 5500 V, and 35 psi) system with online mass spectrometry detection using LIT and QQQ scans and controlled by Analyst 1.6.3 software (SCIEX). After the acquisition of mass spectrometry data, chromatographic peaks for all target compounds were integrated by Multiquant 3.0.3 software (SCIEX) and quantified by using external standard curves.

### 2.4. MeRIP-Seq and RNA-Seq

A total of 25 ug RNA was used before poly(A) selection. Poly(A) RNA was specifically captured with Dynabeads Oligo (dT), then fragmented by using Magnesium RNA Fragmentation Module (NEB, Ipswich, MA, USA). RNA fragments were divided into two groups. One group was incubated with m^6^A antibody (Cat. No. 202003, Synaptic Systems, Göttingen, Germany) in immunoprecipitation (IP) buffer at 4 °C for 2 h to perform m^6^A RNA IP. The other group was used to construct the input samples without IP. IP RNA was reversely transcribed into cDNA by using SuperScript^TM^ II Reverse Transcriptase (Invitrogen, CA, USA). Next, cDNA was applied to synthesize U-labeled second-stranded DNAs with Escherichia coli DNA polymerase I (NEB, Ipswich, MA, USA), RNase H (NEB, Ipswich, MA, USA) and dUTP Solution (Thermo Fisher, Waltham, MA, USA). Following A-tailing, dual-index adapters were ligated, and size selection was performed by using AMPure XP beads. The U-labeled second-stranded DNAs were then digested with heat-labile UDG enzyme (NEB, Ipswich, MA, USA), and the ligated products were amplified by PCR. The sequencing libraries of IP samples (used for MeRIP-seq) and input samples (used for RNA-seq) were subjected to paired-end sequencing (mode PE150) on an Illumina Novaseq^TM^ 6000 platform by LC-Bio Technologies (Hangzhou, China).

### 2.5. Integrated Analysis of MeRIP-Seq and RNA-Seq Bioinformatics Analysis

Raw sequencing data were filtered by using fastp (v0.19.4) to remove adaptors, reads containing poly(A)/poly(G) tails, reads containing > 5% ambiguous bases (N), and low-quality reads. High-quality clean reads were aligned to the sheep reference genome (Ovis_aries, Ensembl v109) by using HISAT2 (v2.2.1). higher read abundance than the input library formed distinct read enrichment regions, which are referred to as peaks. Genome-wide peak calling was performed with the R package exomePeak2 (1.4.0). Identified peaks were annotated by employing ANNOVAR (2013Aug23), and motif analysis was conducted with HOMER (v4.10). Differential m^6^A peaks were identified by using exomePeak2, with thresholds of |log_2_(fold change)| ≥ 1 and FDR < 0.05. Gene expression was quantified with StringTie (v2.2.1) and ballgown, with expression values being reported as fragments per kilobase of transcript per million mapped reads (FPKM). Differential gene expression analysis was performed by using DESeq2, with thresholds of |log_2_(fold change)| ≥ 1 and FDR < 0.05. GO and KEGG analyses were conducted on differentially m^6^A methylated genes (DMGs) and differentially expressed genes (DEGs) through the ClusterProfiler R package. GO terms and KEGG pathways with *p* < 0.05 were considered nominally significant enrichment for supporting exploratory analysis. Candidate genes relevant to DNA repair, apoptosis, and stress responses were prioritized from among those meeting the thresholds of |log(fold change)| ≥ 1 and FDR < 0.05 in both the MeRIP-seq and RNA-seq analyses.

### 2.6. Quantitative Real-Time PCR

Total RNA was extracted from the two groups of testes by using RNA Easy Fast Tissue/Cell Kit (Tiangen, Beijing, China). RNA integrity was assessed by using agarose gel electrophoresis and a Nanodrop 2000 spectrophotometer (Thermo Scientific, MA, USA). RNA samples with OD_260_/_280_ between 1.8 and 2.0 and OD_260_/_230_ higher than 2.0 were considered highly pure. cDNA was synthesized from 1 μg of total RNA by utilizing PrimeScript^TM^ RT reagent Kit with gDNA Eraser (Perfect Real Time) (TaKaRa, Dalian, China) in accordance with the manufacturer’s recommendations after the removal of residual genomic DNA. It was then diluted with DNase-free water 20 times and stored at −80 °C until use. Five genes related to m^6^A modification were detected (Table 1). The qRT-PCR reaction mixtures contained 10 µL of 2× PerfectStart^®^ Green qPCR SuperMix, 0.5 µL of forward primers, 0.5 µL of reverse primers, 5 µL of cDNA, and 4 µL of nuclease-free water. The reaction steps involved predenaturation at 95 °C for 5 min followed by 45 cycles at 95 °C for 5 s and 59 °C for 35 s and were performed by using CFX96 Touch Real-Time PCR Detection System (Bio-Rad, Hercules, CA, USA). Melting curve analysis was conducted to verify the amplification of a single product. The relative quantification of each gene was normalized to RPS18 by using the 2^−ΔΔCt^ method.

### 2.7. Statistical Analysis

The results were evaluated using GraphPad Prism 9.0 (GraphPad Software Inc., San Diego, CA, USA) and presented as mean ± standard error of the mean. Normal parameters were analyzed by Shapiro–Wilk test. Homogeneity of variance was analyzed by Levene’s test. Differences between groups were analyzed by two-tailed Student’s t-tests. *p* < 0.05 was considered statistically significant.

## 3. Results

### 3.1. Histological Observation

**Figure 1:**
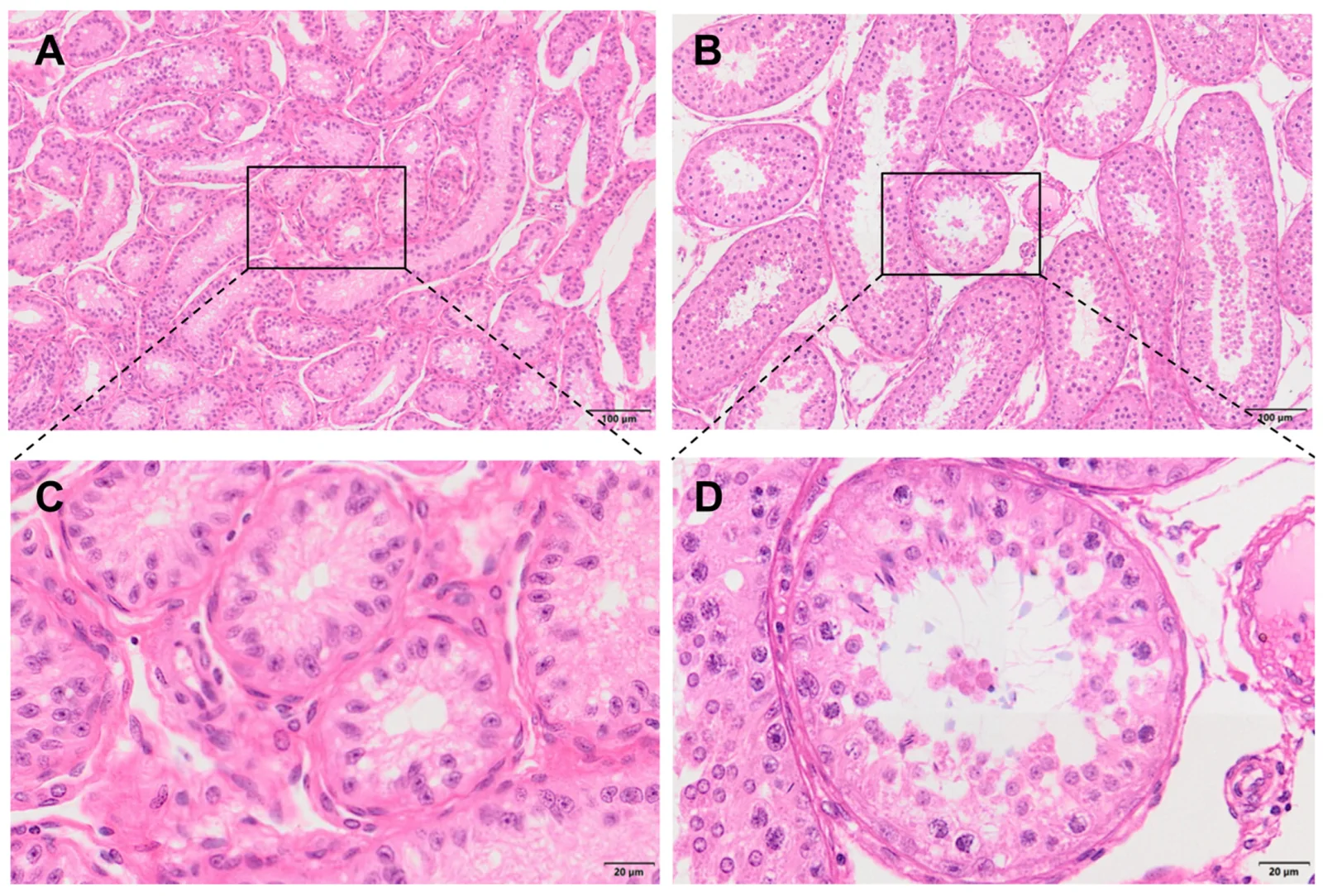
Hematoxylin–eosin staining of cryptorchid (**A**,**C**) and normal (**B**,**D**) testes. Hematoxylin stains cell nuclei blue-purple, whereas eosin stains the cytoplasm and extracellular matrix pink.

Body weight was similar between cryptorchid and normal rams, whereas testicular weight was significantly reduced (*p* < 0.05; Table 2). Cryptorchid testes exhibited markedly reduced spermatogenic cells, whereas normal testes exhibited normal seminiferous epithelium, with morphologically intact sperm present within the seminiferous tubule lumen (Figure 1). The number of Sertoli and Leydig cells and diameter of seminiferous tubules were significantly greater in normal testes than in cryptorchid testes (*p* < 0.05; Table 2).

### 3.2. Global m^6^A Content

**Figure 2:**
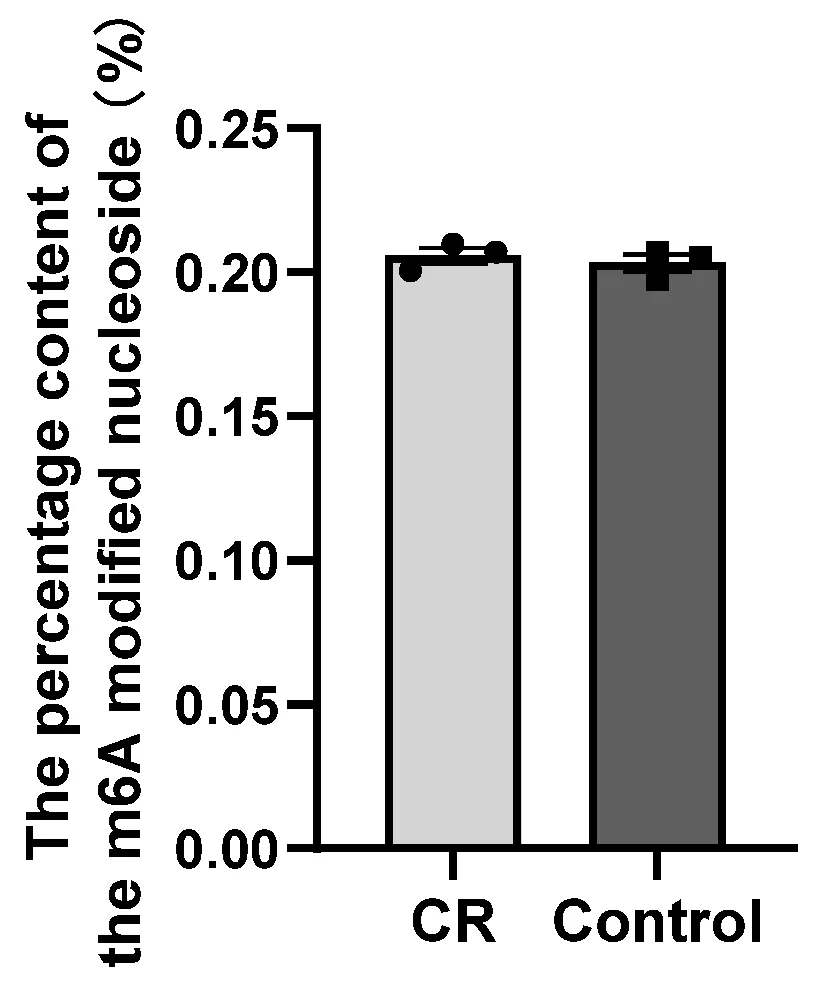
LC–MS/MS quantification of m^6^A levels in testes from cryptorchid (CR) and normal (control) Tianhua mutton sheep.

LC–MS/MS quantification revealed no significant difference in global m^6^A content between groups (*p* > 0.05; Figure 2).

### 3.3. Sequencing Quality Control and Reference Genome Alignment of MeRIP-Seq and RNA-Seq

After quality control, valid bases ranging from 5.18 Gb per sample to 5.78 Gb per sample were obtained. The average Q20 and Q30 base distributions were 98.16% and 96.52%, and the average GC content was 49.94%, indicating high sequencing quality (Table 3).

### 3.4. Methylation Peak Detection and Annotation

**Figure 3:**
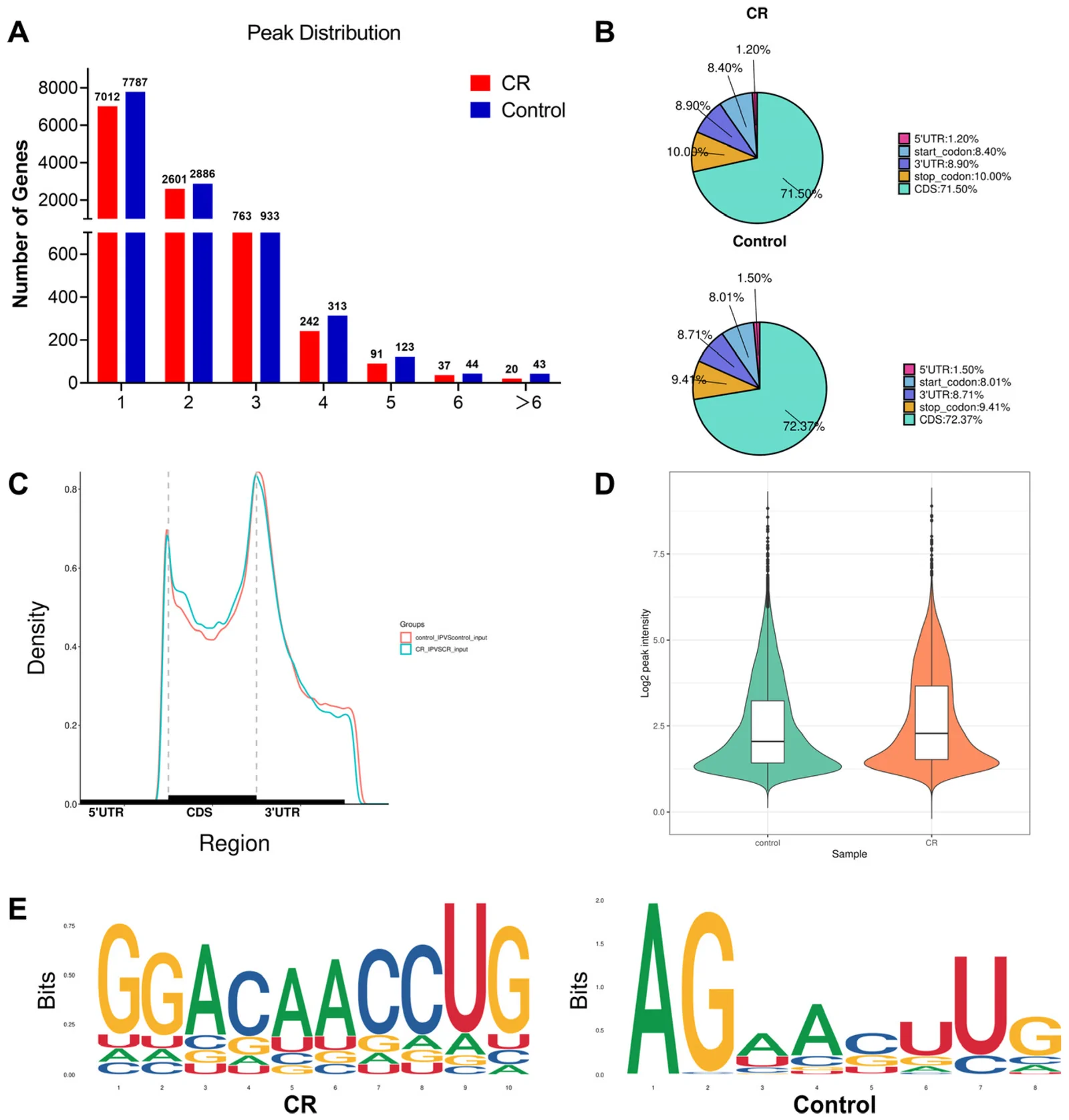
Global m^6^A methylation levels and profiles in testes from cryptorchid (CR) and normal (control) Tianhua mutton sheep. (**A**) Number of m^6^A peaks in the CR and control groups. (**B**) Pie charts demonstrating m^6^A peak distributions in mRNA. (**C**) Metagene plots displaying the regions of m^6^A peaks identified throughout transcripts. Dashed lines represent start and end codons. (**D**) Global m^6^A modification levels by m^6^A-seq. (**E**) Top motifs enriched from m^6^A peaks.

In total, 16,308 and 18,826 m^6^A peaks were detected in the cryptorchidism and control groups, respectively. The genes with only one peak accounted for 65.1% and 64.2% in the cryptorchidism and control groups, respectively (Figure 3A). The majority of the peaks were located in the CDS, followed by stop codons and 3′ UTR (Figure 3B). Peaks were enriched in 3′ UTR near stop codons (Figure 3C). Violin plot analysis of m^6^A peak intensity showed global m^6^A modification levels in the cryptorchidism and control group. However, the difference was not statistically significant (Figure 3D). Motif analysis showed that the identified m^6^A peaks were significantly enriched at the classic RRACH consensus sequence (R = A/G; H = A/C/U), supporting the reliability of the m^6^A profiling data (Figure 3E).

### 3.5. DMG and Functional Enrichment Analyses

**Figure 4:**
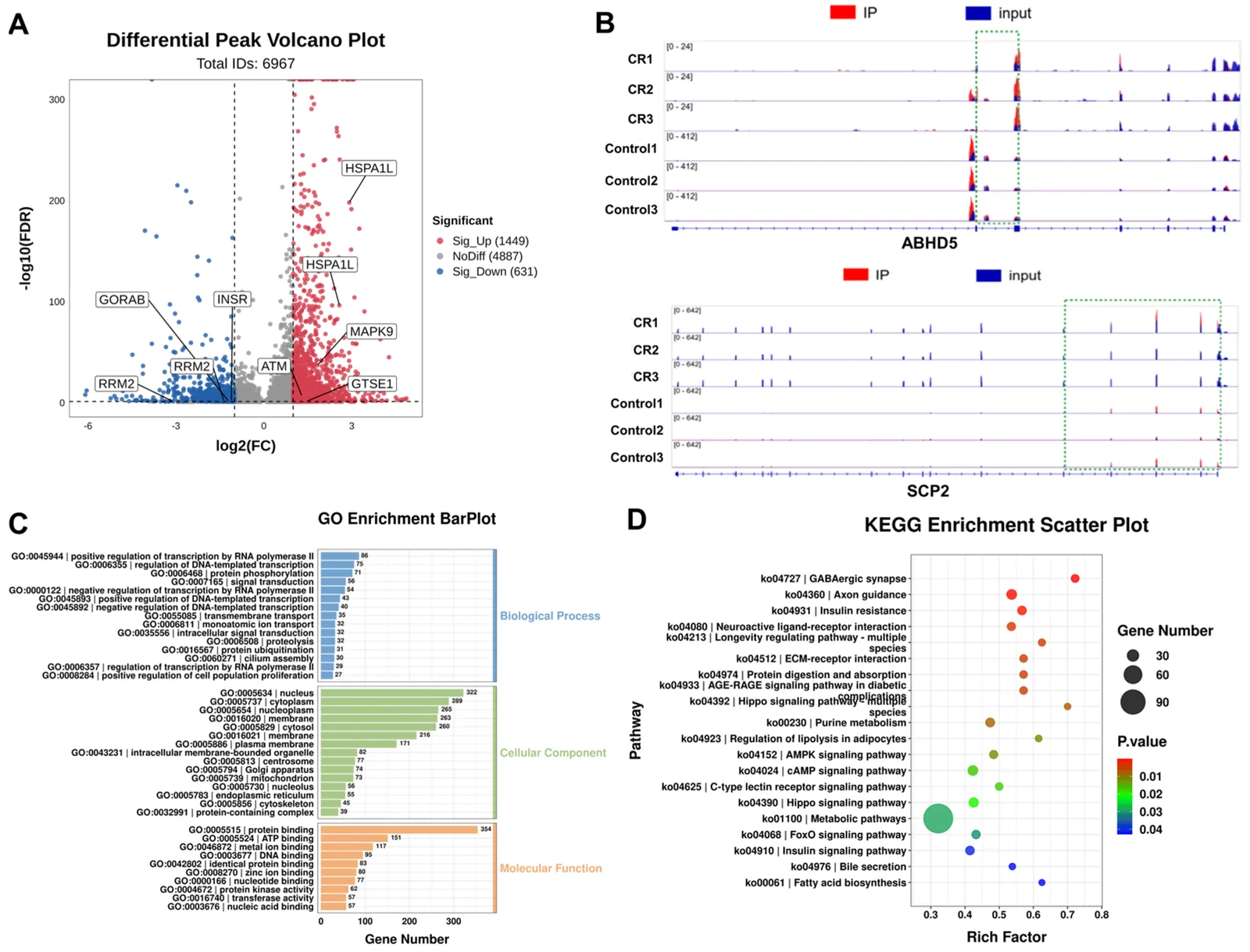
Transcriptome–wide m^6^A analysis of testes from cryptorchid (CR) and normal (control) Tianhua mutton sheep. (**A**) Volcano plots showing m^6^A peaks with significant differential methylation between the CR and control groups. (**B**) Data visualization of differential m^6^A peaks in selected mRNAs (ABHD5 and SCP2) in the CR and control groups. The bottom panel shows the gene structure: blue blocks represent exons, horizontal lines denote introns, and arrows indicate gene orientation. Red and blue peaks represent m^6^A methylation and input signals, respectively. (**C**) GO analysis of differentially methylated genes. (**D**) KEGG enrichment analysis of differentially methylated genes.

Differential peak analysis identified 2080 peaks with significant differential methylation between the cryptorchidism and control groups, including 1449 hypermethylated and 631 hypomethylated peaks in the cryptorchidism group (Figure 4A). Two representative genes, *ABHD5* and *SCP2*, were selected to illustrate the m^6^A methylation pattern. In the cryptorchidism group, *ABHD5* m^6^A peaks were predominantly enriched in the 3′ UTR and exons, whereas *SCP2* m^6^A peaks primarily localized in exons (Figure 4B).

To investigate the effect of m^6^A methylation in differential gene expression between the cryptorchidism and control groups, DMGs were subjected to functional enrichment analysis using the GO and KEGG databases. GO analysis revealed the significant enrichment of 68 biological process terms (the positive/negative regulation of transcription by RNA polymerase II, regulation of DNA-templated transcription, and proteins phosphorylation), 25 cellular component terms (nucleus, nucleoplasm, and cytoplasm), and 27 molecular function terms (protein binding, ATP binding, and metal ion binding) (Figure 4C). KEGG analysis showed that DMGs were enriched in the insulin resistance pathway and the AMPK, Hippo, and FoxO signaling pathway (Figure 4D; Supplementary Table S1).

### 3.6. Overview of Transcriptomic Analysis

**Figure 5:**
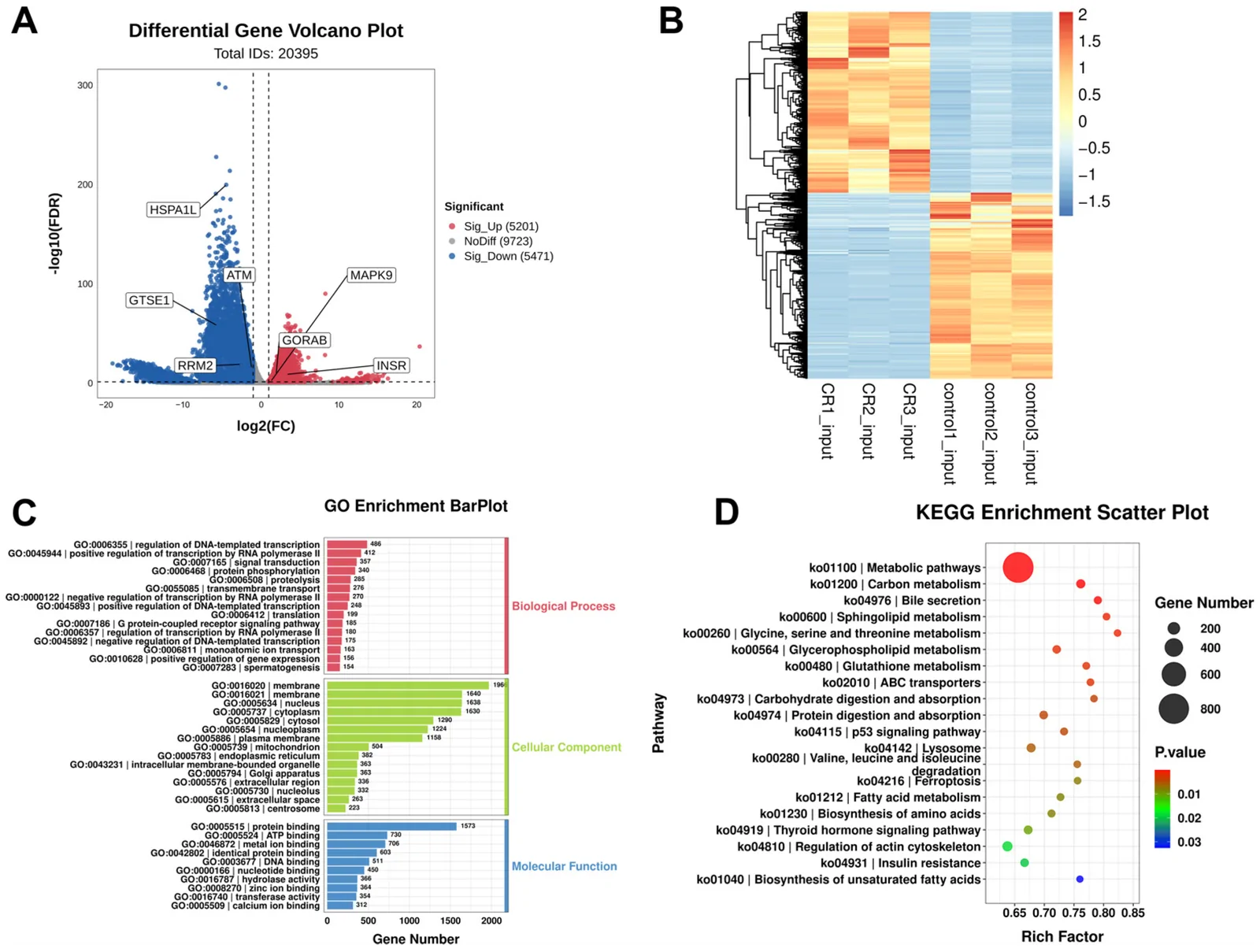
Differentially expressed genes (DEGs) analysis of testes from cryptorchid (CR) and normal (control) Tianhua mutton sheep. (**A**) Volcano plots showing DEGs between the CR and control groups. (**B**) Heatmap plot of all DEGs between the CR and control groups. (**C**) GO analysis of all DEGs. (**D**) KEGG enrichment analysis of all DEGs.

RNA-seq analysis identified 5201 up-regulated and 5471 down-regulated genes in the cryptorchidism group relative to the control group (Figure 5A). The heatmap presented that samples within each group clustered together, with distinct transcriptomic profiles between the cryptorchidism and control groups (Figure 5B). The GO analysis of DEGs revealed that the top enriched terms were similar to those of the DMGs reported in Section 3.5 (Figure 5C). KEGG analysis showed that DEGs were significantly enriched in the p53 signaling pathway (Table 4 and Table S2; Figure 5D).

### 3.7. Integrated Analysis of MeRIP-Seq and RNA-Seq Data

**Figure 6:**
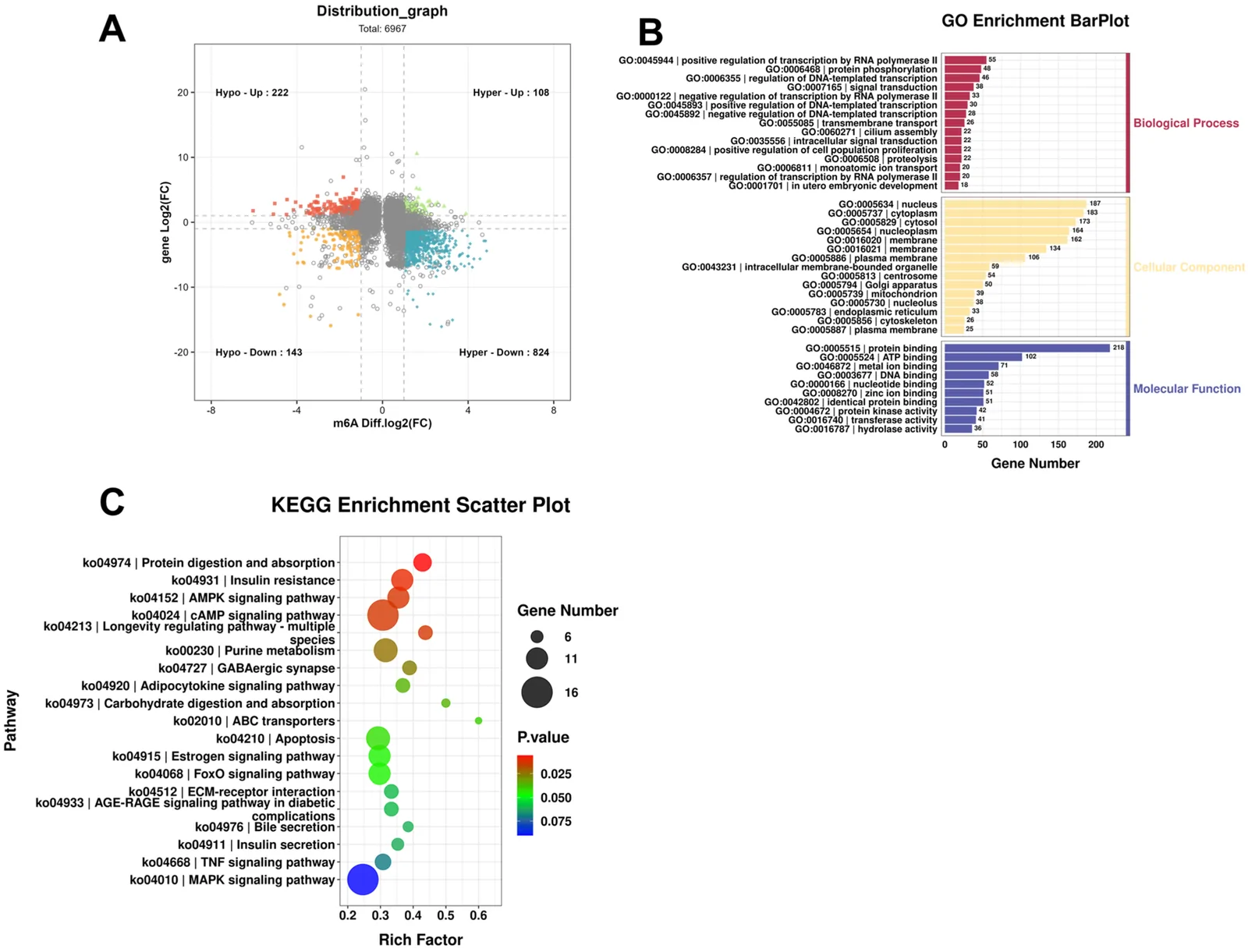
Integrated analysis of m^6^A-seq and RNA-seq data of testes from cryptorchid (CR) and normal (control) Tianhua mutton sheep. (**A**) Four quadrant plots showing differentially expressed genes with differentially methylated m^6^A peaks (|log_2_(fold change)| ≥ 1, FDR < 0.05) between the CR and control groups. Gray circles indicate genes that were not statistically significant. (**B**) GO enrichment analysis of the genes with significant changes in m^6^A and mRNA levels in the CR and control groups. (**C**) KEGG pathway enrichment analysis of the genes with significant changes in m^6^A and mRNA levels in the CR and control groups.

The integrative analysis of MeRIP-seq and RNA-seq data was performed to identify genes exhibiting differential m^6^A modification and altered mRNA expression. Four quadrant plots revealed 1297 DEGs with differentially methylated m^6^A peaks (|diffPeak.log2(foldchange)| ≥ 1, diffPeak.FDR < 0.05 & |diffExp.gene.log2(foldchange)| ≥ 1, diffExp.gene.FDR < 0.05). These DEGs included 108 hypermethylated and up-regulated genes (hyperup), 824 hypermethylated and down-regulated genes (hyperdown), 222 hypomethylated and up-regulated genes (hypoup), and 143 hypomethylated and down-regulated genes (hypodown) (Figure 6A). GO analysis revealed significant enrichment of terms associated with positive/negative regulation of transcription by RNA polymerase II, nucleus, and protein binding terms (Figure 6B). KEGG enrichment analysis identified the signaling pathways associated with differential m^6^A methylation. These pathways included the apoptosis, AMPK, FoxO, TNF, and MAPK signaling pathway (Table 5 and Table S3; Figure 6C).

### 3.8. Expression of m^6^A Regulators

**Figure 7:**
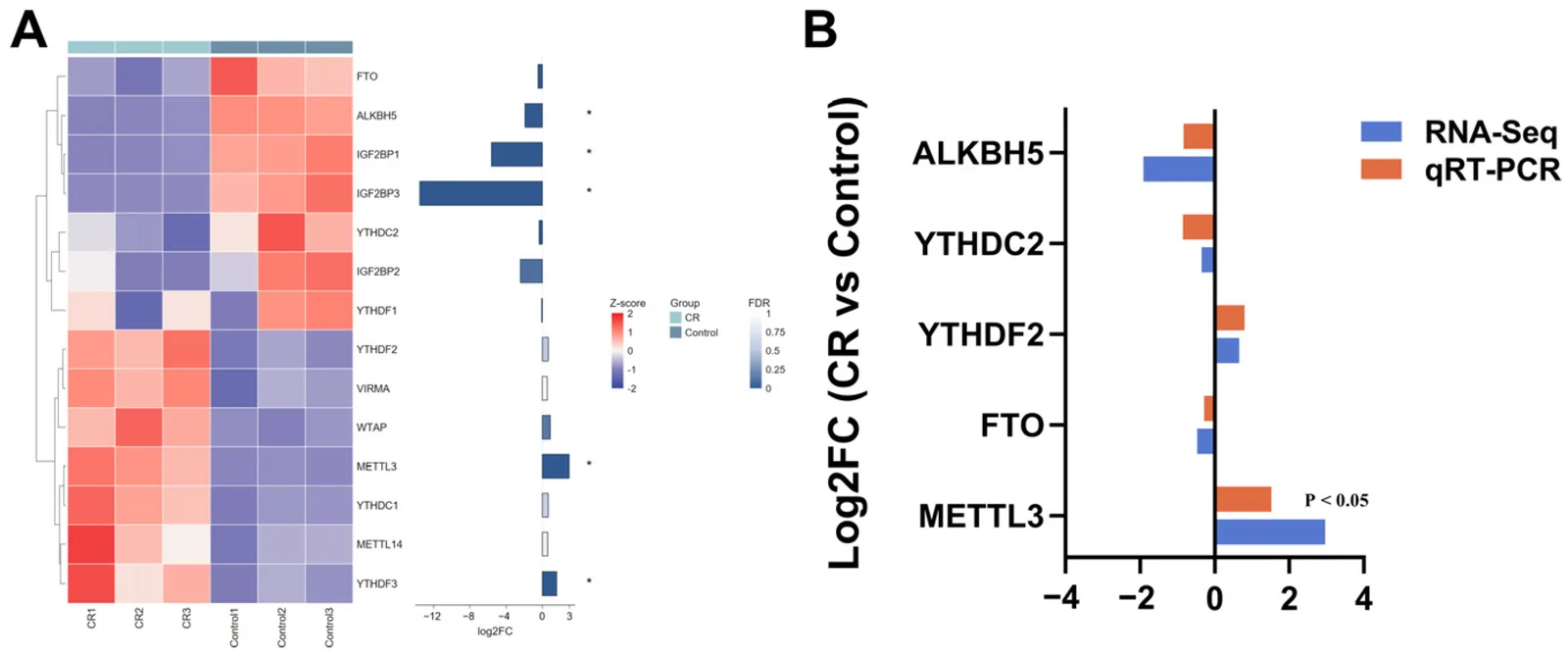
The expression levels of m^6^A regulators. (**A**) Heatmap plot showing the expression levels of m^6^A regulators based on RNA-seq data. (**B**) Comparison of log2(fold change) in the expression of m^6^A regulators between RNA-seq and qRT-PCR. * FDR < 0.05.

Transcriptomic analysis showed that the mRNA expression levels of *ALKBH5*, *IGF2BP1*, and *IGF2BP3* were significantly down-regulated in cryptorchid testes relative to those in normal testes, whereas the expression levels of *METTL3* and *YTHDF3* were significantly up-regulated (FDR < 0.05). The qRT-PCR result exhibited expression patterns consistent with the RNA-seq data (Figure 7B).

## 4. Discussion

In mammals, cryptorchidism is generally understood as a complex reproductive disorder arising from the combined effects of multiple genetic and environmental risk factors. In the present study, cryptorchid testes exhibited narrowed seminiferous tubule diameters, decreased Sertoli and Leydig cell counts, and pronounced depletion of spermatogonia and spermatocyte, indicating spermatogenic arrest. These findings align with previous reports on rats [31], pigs [13], and yaks [32]. Abnormal testicular descent exposes the testis to an unfavorable microenvironment that includes elevated temperature, endocrine disturbance, hypoxia, oxidative stress, and inflammatory stimulation [31]. Thermal stress is an important component of this multifactor damage cascade. Previous studies have established that intra-abdominal hyperthermia disrupts germ cell differentiation into adult dark spermatogonia [9]. In addition, cryptorchidism triggers multiple testicular alterations, including reduced Leydig cell numbers, decreased Sertoli cell nuclear volume, and a 95% reduction in FSH receptor expression in Sertoli cells [33]. These changes impair the development of the spermatogenic cell lineage, exacerbate interstitial fibrosis, and even completely arrest spermatogenesis [31].

Epigenetic regulation, particularly m^6^A modification, coordinates gene expression during testicular development and spermatogenesis [34]. In mammals, m^6^A modification predominantly occurs within conserved RRACH motifs, with prominent enrichment at stop codons and 3′ UTRs [35]. In this study, m^6^A peaks in the testicular tissues of Tianhua mutton sheep predominantly localized to the CDS, stop codons and 3′ UTRs, consistent with the patterns observed in porcine [34] and bovine [26] testes, indicating the tissue specificity and evolutionary conservation of m^6^A distribution. However, cryptorchid testes exhibited slightly altered m^6^A enrichment patterns in CDS regions, 3′ UTRs, and stop codons, suggesting dysregulated posttranscriptional regulation of mRNAs that may affect mRNA stability, translation efficiency, or transcript fate. These alterations may be attributed to the loss of germ cells.

The effects of m^6^A modification on RNA fate depend primarily on peak location and reader type. m^6^A peaks in the 3′ UTR are often associated with the RNA stability. Recognition by YTHDF2 generally promotes mRNA degradation and reduces expression, whereas recognition by IGF2BP1/2/3 enhances mRNA stability and increases expression. The regions near stop codons are canonical sites of m^6^A enrichment. m^6^A modifications in these regions are commonly recognized by readers, such as YTHDF1 and YTHDF3, and are primarily associated with the regulation of translation efficiency. m^6^A modifications within CDSs can regulate ribosome elongation and translation processes, with the direction of these effects depending on the specific reader and cellular context [22]. In the present study, a total of 2080 peaks showing significant differential methylation were identified between the cryptorchidism and control groups. These peaks included 1449 hypermethylated and 631 hypomethylated peaks in the cryptorchidism group. We observed up-regulation of *METTL3* and *YTHDF3*, and down-regulation of *ALKBH5*, *IGF2BP1*, and *IGF2BP3* in cryptorchid testes. m^6^A regulators have been reported to play important roles in maintaining the epitranscriptomic homeostasis required for normal spermatogenesis [36]. The METTL3/METTL14 methyltransferase complex promotes the proliferation and differentiation of spermatogonial stem/progenitor cells during spermatogenesis [37]. The demethylase ALKBH5 negatively regulates the proliferation of immature Sertoli cells by modulating *SOX9* mRNA methylation [34]. *Alkbh5* deficiency in spermatogonia results in aberrantly elevated global m^6^A levels and increased apoptosis of meiotic spermatocytes, ultimately disrupting spermatogenesis [21]. Bisphenol A-induced testicular injury is associated with altered FTO expression, increased apoptosis of testicular cell, and changes in m^6^A methylation patterns [38]. The m^6^A reader protein YTHDC2 is essential for male fertility, and its knockout causing testicular atrophy and spermatogenic arrest [39]. These findings suggest that dysregulated mRNA expression of m^6^A regulators in cryptorchid testes may impair spermatogenesis, although the cell-type specificity and causal roles require further validation.

Our study revealed a distinct transcriptomic profile in the cryptorchid testes of Tianhua mutton sheep. KEGG analysis showed that the DEGs were enriched in the p53 signaling pathways. Previous studies have demonstrated that cryptorchidism induces germ cell apoptosis through the p53 signaling pathway, with p53 expression increasing in a time-dependent manner [40]. Knockout of an m^6^A methyltransferase has been shown to substantially alter gene expression and alternative splicing patterns, thereby modulating the p53 signaling pathway and apoptosis [41]. In this study, we identified several p53 signaling pathway genes with differentially methylated m6A peaks, including *RRM2* (hypodown), *GTSE1* (hyperdown), *ATM* (hyperdown), and *GORAB* (hypoup). RRM2 directly interacts with p53 and catalyzes the synthesis of the deoxyribonucleotides required for DNA repair. *Rrm2* knockdown in theca/interstitial cells reduced DNA synthesis, inhibited proliferation, and promoted early apoptosis [42]. GTSE1 acts as a critical negative regulator of p53 activity, suppressing p53-dependent apoptosis primarily by binding to p53 and promoting its nuclear export and subsequent degradation [43]. ATM is responsible for recognizing DNA double-strand break (DSB) signals and coordinating repair during meiotic homologous recombination. Consequently, *Atm*-deficient male mice are infertile and exhibit pachytene arrest during meiosis prophase I and widespread germ cell apoptosis [44]. Study shows that following its recruitment in response to radiation or oxidative stress, METTL3 can regulate *ATM* expression by modulating its alternative splicing and mRNA decay, thereby contributing to the establishment of cellular stress thresholds [45]. In addition, GORAB participates in Golgi vesicular transport and the maintenance of Golgi structural integrity, while also contributing to cellular stress responses [46]. These results suggest that altered m^6^A methylation may be related to p53-mediated apoptosis and cell cycle arrest.

Integrated analysis of MeRIP-seq and RNA-seq data identified 1297 overlapping genes between DEGs and DMGs. These genes were enriched in stress response pathways, including apoptosis and the AMPK, FOXO, MAPK, and TNF signaling pathway. The AMPK signaling pathway is a critical metabolism and energy stress pathway, which plays a pivotal role in regulating energy balance and male reproductive function [47]. FOXO transcription factors regulate key biological processes, such as metabolic homeostasis, antioxidant responses, apoptosis, and longevity, and the MAPK/ERK signaling pathway affects Sertoli cell proliferation and apoptosis. The TNF signaling pathway has increasingly been recognized as an important inflammatory and stress-responsive pathway in testicular dysfunction. In this study, three genes from stress-response pathways have been reported to be involved in male fertility, were prioritized as candidates: *INSR* (hypoup), *HSPA1L* (hyperdown), and *MAPK9* (hyperup). *INSR* encodes the insulin receptor, which may influence Sertoli cell metabolic support, Leydig cell steroidogenic function, and germ cell survival. The HSPA1L protein is primarily localized to the postacrosomal and midpiece regions of sperm, with reduced expression in low-motility sperm and aged testes [48]. MAPK9, also known as JNK2, is a stress-activated member of the MAPK family and participates in cellular responses to inflammatory stimuli, oxidative stress, and DNA damage, as well as in apoptosis [49]. The elevated mRNA expression of *INSR* and *MAPK9* suggests that cryptorchid testes may undergo metabolic stress, inflammation, and oxidative stress. These candidate genes exhibit concurrent changes in mRNA abundance and m^6^A enrichment, with reported functions in DNA repair, apoptosis, and stress responses. Given that the differential peaks indicate changes in regional m^6^A enrichment and do not establish site-specific modification changes or their causal effects on gene expression, whether these genes contribute to cryptorchidism-associated testicular injury through m^6^A epigenetic modification remains to be verified.

In addition, cryptorchid testes exhibit reduced germ cell numbers [13,14]. Changes in cellular composition may contribute to the observed differences in bulk-tissue gene expression. However, the present data cannot disentangle these contributions, which is a limitation of our study. Future studies should therefore examine differences in the abundance of m^6^A regulator transcripts at single-cell resolution and account for species differences and developmental stage.

## 5. Conclusions

This study compared regional m^6^A enrichment and transcript abundance between normal and cryptorchid sheep testes. The analyses revealed concurrent differences in these profiles and identified candidate genes for further investigation. Since the measurements were obtained from bulk tissue, changes in cellular composition may contribute to the observed patterns. These findings provide an observational resource for future research. However, a causal role for m^6^A regulation in spermatogenic impairment remains to be established.

## Figures and Tables

**Table 1:** Primer sequences used for qRT-PCR.

Gene	Primer Sequence (5’–3’)	Product Size (bp)
*METTL3* XM_004010362.5	F: AGGAACACTGCTTGGTTGGT R: GCCAGGGGACAGTCTTTCAA	143
*FTO* NM_001104931.1	F: GATGGAGTGGCTGACAAGCG R: TGATCAGCGGGTAAAGTCCG	177
*ALKBH5* XM_004023544.5	F: TGACTGTGCTCAGTGGATACG R: TCCCTGTTGTTTCCCGACAG	193
*YTHDC2* XM_015096775.3	F: TACAACGAAAAGGCAGGGCA R: AACTGGGGCTAACAGCTTGG	158
*YTHDF2* XM_027965411.2	F: AGAAAGTGTTAAAAAGGAACGTCA R: TAAACTGCAAGTCTGCAATCGT	172
*RPS18* XM_004018745.3	F: CACTGAGGACGAGGTGGAAC R: CTGTGGGCCCGAATCTTCTT	186

*METTL3* = methyltransferase 3, N6-adenosine-methyltransferase complex catalytic subunit; *FTO* = FTO alpha-ketoglutarate dependent dioxygenase; *ALKBH5* = alkB homolog 5, RNA demethylase; *YTHDC2* = YTH domain-containing protein 2; *YTHDF2* = YTH N6-methyladenosine RNA binding protein F2; *RPS18* = ribosomal protein S18.

**Table 2:** Testicular histomorphometric measurements, right testis weight, and body weight in cryptorchid (CR) and normal (control) Tianhua mutton sheep.

Groups	No. of Sertoli Cells Per Tubule	No. of Leydig Cells Per Triangular Space	Seminiferous Tubule Diameter (μm)	Right Testes Weight (g)	Body Weight (kg)
CR	7.17 ± 0.77 **	12.97 ± 0.71 **	95.16 ± 21.53 **	50.30 ± 12.53 **	54.92 ± 1.86
Control	20.75 ± 2.03	26.10 ± 2.72	178.85 ± 10.64	117.00 ± 2.02	55.07 ± 1.85

** indicates *p* < 0.01.

**Table 3:** Summary of sequencing data and reads alignment statistics from MeRIP-seq and RNA-seq of testes from cryptorchid (CR) and normal (control) Tianhua mutton sheep.

Sample ID	Raw Reads	Raw Bases	Valid Reads	Valid Bases	Valid%	Q20%	Q30%	GC%
CR1_input	37,676,414	5.65 G	37,475,344	5.20 G	91.94	98.38	96.93	49.16
CR2_input	38,843,132	5.83 G	38,633,864	5.36 G	92.08	98.34	96.86	49.39
CR3_input	39,881,032	5.98 G	39,662,486	5.53 G	92.42	98.32	96.81	49.58
Control1_input	39,891,528	5.98 G	39,667,978	5.50 G	91.95	98.37	96.88	50.45
Control2_input	40,082,196	6.01 G	39,848,376	5.53 G	91.91	98.27	96.71	50.26
Control3_input	41,353,786	6.20 G	41,116,894	5.70 G	91.91	98.30	96.76	50.32
CR1_IP	36,648,052	5.50 G	36,344,688	5.18 G	94.18	97.82	95.95	49.68
CR2_IP	37,897,614	5.68 G	37,590,722	5.34 G	93.88	97.86	96.01	49.81
CR3_IP	39,355,080	5.90 G	39,031,224	5.52 G	93.54	97.90	96.07	50.31
Control1_IP	40,048,320	6.01 G	39,702,646	5.67 G	94.38	98.16	96.50	50.17
Control2_IP	40,227,982	6.03 G	39,904,622	5.69 G	94.35	98.07	96.35	50.11
Control3_IP	40,828,412	6.12 G	40,492,374	5.78 G	94.37	98.09	96.38	50.14

**Table 4:** Kyoto encyclopedia of genes and genomes (KEGG) enrichment analysis of DEGs in the p53 signaling pathway in the testes from cryptorchid (CR) and normal (control) Tianhua mutton sheep. Genes with significantly hypermethylated (hyper) or hypomethylated (hypo) m^6^A peaks (FDR < 0.05) are shown in parentheses.

Pathway ID	Pathway Name	Up Regulated Genes	Up Number	Down Regulated Genes	Down Number	*p* Value	FDR
ko04115	p53 signaling pathway	*TP53*, *SESN2*, *CCNG1*, *CASP8*, *BAX*, *GADD45B*, *CCNG2*, *IGFBP3*, *GORAB* (hypo), *CCND2*, *SERPINB5*, *CDK4*, *GADD45G*, *CHEK2*, *CYCS*, *TSC*, *RRM2B*, *THBS1*, *MDM2*, *CASP9*, *GADD45A*, *CCND3*, *LOC10110318*	23	*PPM1D*, *GTSE1* (hyper), *CCNB1*, *CD82*, *CDK1*, *CCNB2*, *RCHY1*, *SESN3*, *CCNE2*, *ATR*, *RRM2* (hypo), *LOC101103784*, *PTEN*, *ATM* (hyper), *CDK2*, *AIFM2*, *LOC101102964*, *CCNE1*, *CDK6*, *TP73*, *ZMAT3*	21	<0.01	0.09

**Table 5:** Kyoto encyclopedia of genes and genomes (KEGG) enrichment analysis of the integrated analysis of the differentially expressed genes with differentially methylated m^6^A peaks associated with apoptosis and stress response in testes from cryptorchid (CR) and normal (control) Tianhua mutton sheep.

Pathway ID	Pathway Name	Hyper-Up Genes	Hyper-Down Genes	Hypo-Up Genes	Hypo-Down Genes	Both m^6^A Genes	*p* Value	FDR
ko04152	AMPK signaling pathway	-	*PPP2R2B*, *TBC1D1*, *PPARGC1A*, *HMGCR*, *PRKAA1*, *PPP2R3C*, *PDPK1*	*EEF2*, *INSR*, *GYS1*	*G6PC3*	-	0.01	0.64
ko04210	Apoptosis	*MAPK9*	*TUBA4A*, *TUBA8*, *ATM*, *PDPK1*	*MAP3K14*, *NFKBIA*, *CASP7*, *XIAP*	*LMNB2*	*PARP4* (gene: up), *BPHL* (gene: up)	0.04	0.70
ko04068	FoxO signaling pathway	*MAPK9*	*SOS2*, *ATM*, *PRKAA1*, *PLK4*, *PDPK1*, *PLK1*	*INSR*, *HOMER1*	*G6PC3*, *TGFB1*	-	0.05	0.70
ko04668	TNF signaling pathway	*MAPK9*	*BAG4*	*ICAM1*, *MAP3K14*, *NFKBIA*, *RPS6KA4*, *CASP7*	-	*BPHL* (gene: up)	0.07	0.70
ko04010	MAPK signaling pathway	*MAPK9*	*MECOM*, *PPM1A*, *HSPA2*, *SOS2*, *ELK4*, *HSPA1L*	*MAP3K12*, *INSR*, *MAP3K14*, *HSPA8*, *RPS6KA4*, *FGFR1*	*TGFB1*	*CACNA1C* (gene: down), *NFATC3* (gene: down)	0.09	0.70

## Data Availability

The raw sequence data reported in this paper have been deposited in the Genome Sequence Archive (Genomics, Proteomics & Bioinformatics 2025) in National Genomics Data Center (Nucleic Acids Res 2025), China National Center for Bioinformation/Beijing Institute of Genomics, Chinese Academy of Sciences (GSA: CRA049933) that are publicly accessible at https://ngdc.cncb.ac.cn/gsa (accessed on 12 September 2026).

## Supplementary Materials

The following supporting information can be downloaded at: https://www.mdpi.com/article/10.3390/ani16213460/s1, Table S1: Diffpeak KEGG enrichment result; Table S2: DEGs KEGG enrichment result; Table S3: Integrated analysis KEGG enrichment result.

## References

[1] Li D., Liu Z., Duan X., Wang C., Chen Z., Zhang M., Li X., Ma Y. (2024). Rumen development of Tianhua mutton sheep was better than that of Gansu Alpine Fine Wool sheep under grazing conditions. Animals.

[2] Rodprasert W., Virtanen H.E., Mäkelä J.A., Toppari J. (2019). Hypogonadism and cryptorchidism. Front. Endocrinol..

[3] Nassif C., Mara L., Chessa F., Cossu I., Gallus M., Melis F., Cannas A., Dattena M. (2026). Cryptorchidism in Sarda sheep: Incidence, morphology, ultrasonography and behavioral insights. Animals.

[4] Ott R.S., Memon M.A. (1980). Breeding soundness examinations of rams and bucks: A review. Theriogenology.

[5] Amann R.P., Veeramachaneni D.N.R. (2007). Cryptorchidism in common eutherian mammals. Reproduction.

[6] Smith K.C., Brown P.J., Barr F.J., Parkinson T.J. (2012). Cryptorchidism in sheep: A clinical and abattoir survey in the United Kingdom. Open J. Vet. Med..

[7] Lainas T., Deligiannis K. (2002). Cryptorchidism in the Karagouniko breed of sheep and its economic consequences. J. Hell. Vet. Med. Soc..

[8] Greber D., Doherr M., Drögemüller C., Steiner A. (2013). Occurrence of congenital disorders in Swiss sheep. Acta Vet. Scand..

[9] Hutson J.M., Southwell B.R., Li R., Lie G., Ismail K., Harisis G., Chen N. (2013). The regulation of testicular descent and the effects of cryptorchidism. Endocr. Rev..

[10] Gao Y., Xi S., Cai B., Wu T., Wang Q., Kalds P., Huang S., Wang Y., Han S., Pan M. (2025). Sheep with partial RXFP2 knockout exhibit normal horn phenotype but unilateral cryptorchidism. J. Integr. Agric..

[11] Boisen K.A., Kaleva M., Main K.M., Virtanen H.E., Haavisto A.M., Schmidt I.M., Chellakooty M., Damgaard I.N., Mau C., Reunanen M. (2004). Difference in prevalence of congenital cryptorchidism in infants between two Nordic countries. Lancet.

[12] Ivell R. (2007). Lifestyle impact and the biology of the human scrotum. Reprod. Biol. Endocrinol..

[13] Fan X., Liu Y., Yue M., Yue W., Ren G., Zhang J., Zhang X., He J. (2021). Effect of cryptorchidism on the histomorphometry, proliferation, apoptosis, and autophagy in boar testes. Animals.

[14] Wang X., Liu Q., Zhuang Z., Cheng J., Zhang W., Jiang Q., Guo Y., Li R., Lu X., Cui L. (2024). Decoding the pathogenesis of spermatogenic failure in cryptorchidism through single-cell transcriptomic profiling. Cell Rep. Med..

[15] Keel B.A., Zalesky D.D., Sohaili I., Schanbacher B.D., Grotjan H.E. (1994). Heterogeneity of gonadotropins and levels of uncombined luteinizing hormone subunits in pituitaries of cryptorchid rams. J. Androl..

[16] Yin Y., DeWolf W.C., Morgentaler A. (1998). Experimental cryptorchidism induces testicular germ cell apoptosis by p53-dependent and -independent pathways in mice. Biol. Reprod..

[17] Wang X., Feng J., Xue Y., Guan Z., Zhang D., Liu Z., Gong Z., Wang Q., Huang J., Tang C. (2016). Structural basis of N^6^-adenosine methylation by the METTL3–METTL14 complex. Nature.

[18] Ping X.-L., Sun B.-F., Wang L., Xiao W., Yang X., Wang W.-J., Adhikari S., Shi Y., Lv Y., Chen Y.-S. (2014). Mammalian WTAP is a regulatory subunit of the RNA N^6^-methyladenosine methyltransferase. Cell Res..

[19] Pendleton K.E., Chen B., Liu K., Hunter O.V., Xie Y., Tu B.P., Conrad N.K. (2017). The U6 snRNA m^6^A methyltransferase METTL16 regulates SAM synthetase intron retention. Cell.

[20] Jia G., Fu Y., Zhao X., Dai Q., Zheng G., Yang Y., Yi C., Lindahl T., Pan T., Yang Y.-G. (2011). N^6^-Methyladenosine in nuclear RNA is a major substrate of the obesity-associated FTO. Nat. Chem. Biol..

[21] Zheng G., Dahl J.A., Niu Y., Fedorcsak P., Huang C.M., Li C.J., Vågbø C.B., Shi Y., Wang W.L., Song S.H. (2013). ALKBH5 is a mammalian RNA demethylase that impacts RNA metabolism and mouse fertility. Mol. Cell.

[22] Yang Y., Hsu P.J., Chen Y.S., Yang Y.G. (2018). Dynamic transcriptomic m^6^A decoration: Writers, erasers, readers and functions in RNA metabolism. Cell Res..

[23] Wang X., Lu Z., Gomez A., Hon G.C., Yue Y., Han D., Fu Y., Parisien M., Dai Q., Jia G. (2014). N^6^-methyladenosine-dependent regulation of messenger RNA stability. Nature.

[24] Huang H., Weng H., Sun W., Qin X., Shi H., Wu H., Zhao B.S., Mesquita A., Liu C., Yuan C.L. (2018). Recognition of RNA N^6^-methyladenosine by IGF2BP proteins enhances mRNA stability and translation. Nat. Cell Biol..

[25] Zhao T.X., Wang J.K., Shen L.J., Long C.L., Liu B., Wei Y., Han L.D., Wei Y.X., Wu S.D., Wei G.H. (2020). Increased m^6^A RNA modification is related to the inhibition of the Nrf2-mediated antioxidant response in di-(2-ethylhexyl) phthalate-induced prepubertal testicular injury. Environ. Pollut..

[26] Liu S.H., Ma X.Y., Yue T.T., Wang Z.C., Qi K.L., Li J.C., Lin F., Rushdi H.E., Gao Y.Y., Fu T. (2021). Transcriptome-wide m^6^A analysis provides novel insights into testicular development and spermatogenesis in Xia-Nan cattle. Front. Cell Dev. Biol..

[27] Liu Y., Chen Y., Fan J., Min Z., Liu X., Mao Z., Chen A., Liu M., Xia R., Yang L. (2025). IGF2BP1 stabilizes Akt2 mRNA to promote glucose metabolism and maintain spermatogonial proliferation. Reproduction.

[28] Hadziselimovic F., Hadziselimovic N.O., Demougin P., Oakeley E.J. (2011). Testicular gene expression in cryptorchid boys at risk of azoospermia. Sex. Dev..

[29] Liu J., Li W., Weng X., Yue X., Li F. (2022). Composition of fatty acids and localization of SREBP1 and ELOVL2 genes in cauda epididymides of Hu sheep with different fertility. Animals.

[30] Myers R.B., Abney T.O. (1990). Testosterone and androstanediol production by regenerating Leydig cells in the ethylene dimethane sulphonate-treated mature rat. Int. J. Androl..

[31] Wang X., Deng F., Chen Y., Liu X., Li D., Tang X., Lai H., Li Q., Fu W., Liu G. (2025). Developmental phase-specific molecular signatures and signaling pathways in cryptorchidism-induced testicular damage. Biomolecules.

[32] Yang D., Yuan L., Zeng J., Qi Y., Ma L., Li H., Lv J., Chen Y. (2025). Comparative proteomic and transcriptomic analysis of testicular tissue of yaks with or without cryptorchidism. Theriogenology.

[33] Barenton B., Blanc M.R., Caraty A., Hochereau-de Reviers M.T., Perreau C., Saumande J. (1982). Effect of cryptorchidism in the ram: Changes in the concentrations of testosterone and estradiol and receptors for LH and FSH in the testis, and its histology. Mol. Cell. Endocrinol..

[34] Chen C., Tang X., Yan S., Yang A., Xiang J., Deng Y., Yin Y., Chen B., Gu J. (2023). Comprehensive analysis of the transcriptome-wide m^6^A methylome in Shaziling pig testicular development. Int. J. Mol. Sci..

[35] Ke S., Alemu E.A., Mertens C., Gantman E.C., Fak J.J., Mele A., Haripal B., Zucker-Scharff I., Moore M.J., Park C.Y. (2015). A majority of m^6^A residues are in the last exons, allowing the potential for 3′ UTR regulation. Genes Dev..

[36] Zhao Z., Su J. (2026). m^6^A RNA methylation orchestrates spermatogenesis: Decoding the synergistic regulatory network of signaling pathways and ubiquitination modifications. J. Assist. Reprod. Genet..

[37] Lin Z., Hsu P.J., Xing X., Fang J., Lu Z., Zou Q., Zhang K.J., Zhang X., Zhou Y., Zhang T. (2017). Mettl3-/Mettl14-mediated mRNA N^6^-methyladenosine modulates murine spermatogenesis. Cell Res..

[38] Ling Y., Huang X., Li A., Zhang J., Chen J., Ren J., Liu Y., Xie M. (2023). Bisphenol A exposure induces testicular oxidative damage via FTO/m^6^A/Nrf2 axis during postnatal development in mice. J. Appl. Toxicol..

[39] Hsu P.J., Zhu Y., Ma H., Guo Y., Shi X., Liu Y., Qi M., Lu Z., Shi H., Wang J. (2017). Ythdc2 is an N^6^-methyladenosine binding protein that regulates mammalian spermatogenesis. Cell Res..

[40] Absalan F., Movahedin M., Mowla S.J. (2010). Germ cell apoptosis induced by experimental cryptorchidism is mediated by molecular pathways in mouse testis. Andrologia.

[41] Dominissini D., Moshitch-Moshkovitz S., Schwartz S., Salmon-Divon M., Ungar L., Osenberg S., Cesarkas K., Jacob-Hirsch J., Amariglio N., Kupiec M. (2012). Topology of the human and mouse m^6^A RNA methylomes revealed by m^6^A-seq. Nature.

[42] Sa R., Zhang X., Shi K., Wang B., Yu H. (2026). Decreased expression of ribonucleotide reductase M2 accelerates ovarian aging by suppressing cell proliferation and inducing DNA damage in mouse theca-interstitial cells. J. Ovarian Res..

[43] Monte M., Benetti R., Buscemi G., Sandy P., Del Sal G., Schneider C. (2003). The cell cycle-regulated protein human GTSE-1 controls DNA damage-induced apoptosis by affecting p53 function. J. Biol. Chem..

[44] Barlow C., Liyanage M., Moens P.B., Tarsounas M., Nagashima K., Brown K., Rottinghaus S., Jackson S.P., Tagle D., Ried T. (1998). Atm deficiency results in severe meiotic disruption as early as leptonema of prophase I. Development.

[45] Zhang X., Liu P., Zheng X., Wang J., Peng Q., Li Z., Wei L., Liu C., Wu Y., Wen Y. (2021). N^6^-methyladenosine regulates ATM expression and downstream signaling. J. Cancer.

[46] Witkos T.M., Chan W.L., Joensuu M., Rhiel M., Pallister E., Thomas-Oates J., Mould A.P., Mironov A.A., Biot C., Guérardel Y. (2019). GORAB scaffolds COPI at the trans-Golgi for efficient enzyme recycling and correct protein glycosylation. Nat. Commun..

[47] Rahman M.A., Harrath A.H., Jalouli M., Choi J., Choi M., Park S., Kim H.J., Rani A., Akter S., Park M.N. (2026). Targeting AMPK networks for male reproductive health: Mechanisms and emerging therapies. Cells.

[48] Liu X., Teng Z., Wang Z., Zhu P., Song Z., Liu F. (2022). Expressions of HSPA1L and HSPA9 are associated with poor sperm quality of low-motility spermatozoa in fertile men. Andrologia.

[49] Davis R.J. (2000). Signal transduction by the JNK group of MAP kinases. Cell.

